# High Nucleosome Occupancy Is Encoded at Human Regulatory Sequences

**DOI:** 10.1371/journal.pone.0009129

**Published:** 2010-02-09

**Authors:** Desiree Tillo, Noam Kaplan, Irene K. Moore, Yvonne Fondufe-Mittendorf, Andrea J. Gossett, Yair Field, Jason D. Lieb, Jonathan Widom, Eran Segal, Timothy R. Hughes

**Affiliations:** 1 Department of Molecular Genetics, University of Toronto, Toronto, Canada; 2 Department of Computer Science and Applied Mathematics, Weizmann Institute of Science, Rehovot, Israel; 3 Department of Biochemistry, Molecular Biology, and Cell Biology, Northwestern University, Evanston, Illinois, United States of America; 4 Department of Biology, University of North Carolina at Chapel Hill, Chapel Hill, North Carolina, United States of America; University of Pennsylvania, United States of America

## Abstract

Active eukaryotic regulatory sites are characterized by open chromatin, and yeast promoters and transcription factor binding sites (TFBSs) typically have low intrinsic nucleosome occupancy. Here, we show that in contrast to yeast, DNA at human promoters, enhancers, and TFBSs generally encodes high intrinsic nucleosome occupancy. In most cases we examined, these elements also have high experimentally measured nucleosome occupancy *in vivo*. These regions typically have high G+C content, which correlates positively with intrinsic nucleosome occupancy, and are depleted for nucleosome-excluding poly-A sequences. We propose that high nucleosome preference is directly encoded at regulatory sequences in the human genome to restrict access to regulatory information that will ultimately be utilized in only a subset of differentiated cells.

## Introduction

Active regulatory sequences are generally thought to be depleted of nucleosomes, presumably due to steric constraints between nucleosomes and most other DNA-binding proteins, such as transcription factors (TFs). In the yeast *S. cerevisiae*, studies examining the relative incorporation of genomic DNA into nucleosomes *in vitro* have demonstrated that nucleosome depletion at many promoters is to a large extent programmed into the DNA sequence [1], [2]. Regulatory regions in human are typically cell-type-specific [3], however, suggesting that the chromatin state may not be easily encoded directly in the DNA sequence, which does not vary between cell types. The mechanisms by which cell-type specific regulatory elements are specified are poorly understood, but it is reasonable to assume that any mechanism involves interplay between cell-type specific trans-acting factors [4], [5] and the hardwired intrinsic nucleosome-formation preferences of DNA sequences [1].

Here, we apply a computational model of intrinsic nucleosome sequence preference [1] to the human genome. We show that *in vivo* occupancy positively and significantly correlates with intrinsic nucleosome occupancy, indicating that intrinsic histone-DNA sequence preferences play a role in dictating nucleosome arrangement *in vivo*. However, unlike yeast, regulatory sequences in human have higher than average intrinsic nucleosome occupancy, suggesting that restricted access to cell-type specific regulatory DNA is encoded directly in the genomes of complex organisms. We show that this difference is associated with local variations in base composition (G+C content), which correlates with both nucleosome occupancy and regulatory function, as well as the probability of rigid, nucleosome-excluding polyA-like sequences [6], [7]. We suggest possible implications of these overlapping signals in determining chromatin structure and mechanisms of gene regulation.

## Results

Based on the major role that intrinsic histone-DNA preferences play in determining *in vivo* nucleosome occupancy in yeast [1], [2], we speculated that DNA sequence may influence human nucleosome occupancy. We used a model of nucleosome sequence preferences we described previously [1] to compare intrinsic (i.e. DNA-encoded) occupancy with experimentally determined nucleosome occupancy in CD4+ T-cells [8]. Our model is based on the relative preference of chicken histones to assemble on yeast genomic DNA *in vitro*, and, in cross-validation, can predict nucleosome occupancy with an accuracy rivalling that of experimental reproducibility (R = 0.89 vs. R = 0.92 base-by-base correlation for replicate experiments) [1]. The model also correlates well with *in vivo* nucleosome occupancy in yeast (R = 0.75) and *C. elegans* (R = 0.60), as well as *in vitro* histone-DNA affinity of synthetic oligonucleotides (R = 0.45–0.51) [1], [9], indicating that, despite being derived from yeast sequences assembled into nucleosomes *in vitro*, the model is broadly applicable to unrelated genomes as well as artificial sequence.

**Figure 1:**
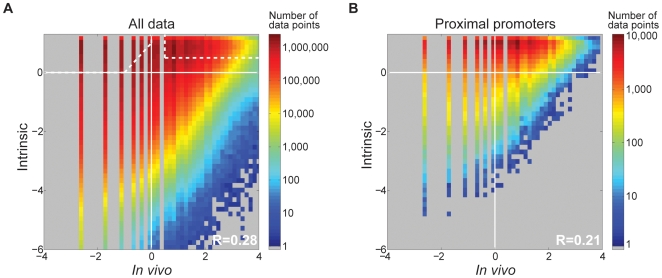
Intrinsic nucleosome occupancy versus *in vivo* nucleosome occupancy in human CD4+ T-cells. Values are on a log_2_ scale, comparing model score [1] vs. *in vivo* occupancy [8] at individual bases across (**A**) the human genome and (**B**) proximal promoters. Pearson correlation is shown. The Spearman P-value is less than 2.2×10^−308^. Quantal behaviour in regions of low nucleosome occupancy is due to sequences that have a low number of reads [8]. The white dashed borders are referred to in the text. Regions of the graph with no data points are shown in gray.

We found that the model scores (hereafter referred to as intrinsic nucleosome occupancy) correlate significantly with *in vivo* nucleosome occupancy in CD4+ T-cells [8] (R = 0.28; **Figure 1A**; range of R is 0.20–0.33 per chromosome). On the basis of Spearman correlation, base-by-base, we calculate P<2.2×10^−308^ over the full genome. To gauge the significance of the correlation on a smaller number of independent loci, we randomly selected 1,000 positions from each chromosome, none of which are within 150 bases of each other, and obtained P-values between 8.2×10^−8^ and 2.2×10^−308^. Thus, there is a significant relationship between intrinsic and *in vivo* nucleosome occupancy, but intrinsic occupancy explains only a minority of *in vivo* nucleosome occupancy.

To gain further insight to the relationship between intrinsic and *in vivo* nucleosome occupancy, we examined **Figure 1A** manually. It is particularly striking that there are very few sequences that have low intrinsic nucleosome occupancy, but high *in vivo* nucleosome occupancy, while there are many sequences with both low intrinsic and low *in vivo* occupancy. This is indicated by the scarcity of points in the lower right portion of the plot in **Figure 1A**, relative to the lower left. This result strongly supports the efficacy of our intrinsic nucleosome occupancy model in human. In contrast, there appear to be many sequences in the upper left portion of **Figure 1A**, indicative of loci with high intrinsic nucleosome occupancy, but low *in vivo* nucleosome occupancy. This observation is consistent with the fact that *trans*-acting factors, such as CTCF [10], can exclude nucleosomes from their binding sites. However, the proportion in the upper left is not as great as the proportion in the upper right, indicating that many human sequences have both high intrinsic and high *in vivo* nucleosome occupancy (for example, the boxes with dotted lines in **Figure 1A** represent 16.6% and 18.0% of the genome, respectively), further underscoring the contribution of intrinsic nucleosome occupancy to nucleosome occupancy *in vivo*.

**Figure 2:**
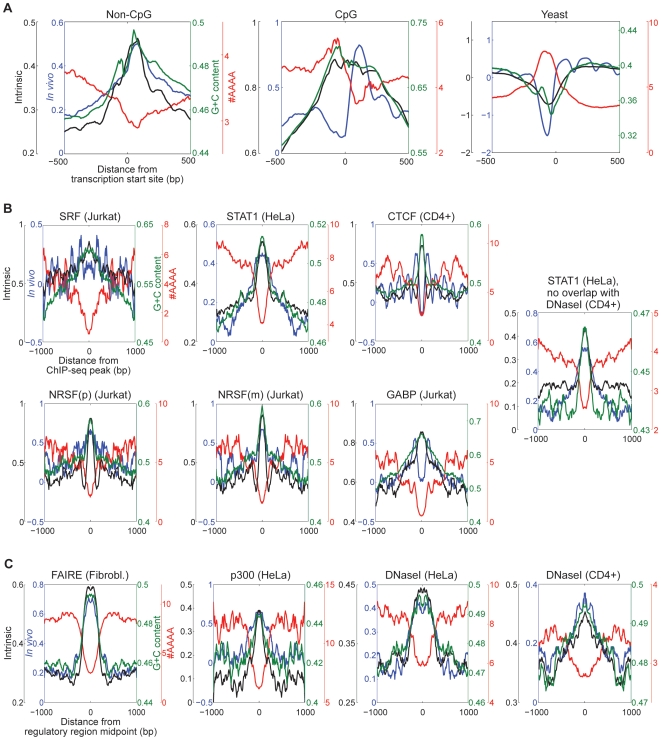
Average profiles of intrinsic nucleosome occupancy, *in vivo* nucleosome occupancy, G+C content and frequency of poly-A (AAAA) sequences in human and yeast promoters, TF binding sites and putative non-promoter regulatory regions. (**A**) Average profiles of 20,286 non-CpG promoters, 11,757 CpG promoters, and 5,015 yeast promoters. (**B**) Experimentally determined transcription factor binding sites. (**C**) Putative regulatory regions. Sequences are defined by the studies indicated in the text and Methods. The average nucleosome occupancy at each base, relative to the center of the binding site or putative regulatory region, is from Schones et al. [8]. Proportion G+C and frequency of the 4-mer “AAAA” are calculated in 150 base windows. Note that vertical axes are different between different panels; they are adjusted to display the full range for each trace in each panel.

In yeast, there is a strong bias for promoters and transcription factor binding sites (TFBS) to be found in locations that have low intrinsic nucleosome occupancy [1]. We therefore examined the average intrinsic nucleosome occupancy of several types of human regulatory sequences, including promoters (**Figure 1B** **and** **2A**), TFBS (**Figure 2B**) [11], [12], [13] and non-promoter regions associated with indicators of either open chromatin (FAIRE [3] and DNaseI hypersensitivity [14], [15]) or enhancer function (p300 association) [14] (**Figure 2C**). In all cases, these regions displayed higher than average intrinsic nucleosome occupancy (black traces in **Figure 2**), and in nearly all cases also displayed higher than average *in vivo* nucleosome occupancy (blue traces in **Figure 2**), rather than lower, as is the case in yeast (**Figure 2A**, rightmost plot). Indeed, if we use the same regions (dashed boxes) in **Figure 1B** (promoters) as described above for **Figure 1A** (all sequences), 22.9% of the data points in 1B are in the upper left (vs. 16.6%) and 33.5% of the data points are in the upper right (vs. 18.0%), i.e. promoter sequences are almost two-fold more likely than the genome average to have both high intrinsic and high *in vivo* nucleosome occupancy. The exceptions to the overall correlation between intrinsic and *in vivo* nucleosome occupancy at regulatory regions are the strong nucleosome depletion just upstream of the transcription start site (TSS) in CpG promoters *in vivo* (**Figure 2A**, center), which is presumably caused by RNA Pol II and associated factors that preferentially associate with CpG promoters [8], [16]; CTCF binding sites that were ascertained in CD4+ cells (the same cell type in which the nucleosome occupancy map was made) (**Figure 2B**); and, to a lesser extent, GABP binding sites determined in Jurkat cells (immortalized T-lymphocytes), consistent with the potential role of GABP as a ubiquitous general regulator [17], [18] (**Figure 2B**).

One potential explanation for the high *in vivo* occupancy of regulatory regions could be measurement bias in the *in vivo* nucleosome occupancy data: since open chromatin is by definition more accessible, it is possible that more nucleosome reads are obtained from regions of open chromatin because these nucleosomes are more accessible to the micrococcal nuclease used to prepare nucleosomes. However, the *in vivo* data is clearly capturing previously-described reductions in nucleosome occupancy at CpG-containing promoters and CTCF sites [8], [10], yet these reductions are not observed at many other sites, including those that have similar G+C content (**Figure 2A, B**). Moreover, high *in vivo* occupancy is observed even at loci that are not recovered as open chromatin in CD4+ cells; for example, STAT1 binding sites in HeLa cells which are not in DNaseI-hypersensitive regions in CD4+ cells still display both high intrinsic nucleosome occupancy and high *in vivo* nucleosome occupancy in CD4+ cells (**Figure 2B**, rightmost panel). Thus, the *in vivo* nucleosome occupancy profiles are not simply measuring open chromatin status.

There are previous indications that G+C content has a strong relationship to nucleosome occupancy in yeast and *C. elegans* [9], [19], [20], and also to nucleosome occupancy at human exons [21]. In a recent analysis we have shown that G+C content also correlates highly with intrinsic nucleosome occupancy and with our model of intrinsic occupancy [22], presumably because it both reduces the frequency of rigid poly-A-like sequences and simultaneously increases the overall capacity for the DNA to bend and twist. This conclusion cannot be accounted for by G+C biases in short-read sequencing [23] used to make some nucleosome maps, because the correlation also holds for data sets created using microarrays as a readout [1], [20], and for a data set that was normalized to sequencing counts for naked genomic DNA [9]. In human, many regulatory sequences, including promoters, tend to have high G+C content [24], and, as predicted by the relationship between nucleosome preferences and base composition, nucleosome occupancy at human promoters and other regulatory sites *in vivo* correlates with G+C content (green traces in **Figure 2**). Regulatory sequences are also depleted for well-established nucleosome-excluding poly-A-like sequences (red lines in **Figure 2**) [6], [25]. We note that in these graphs frequency of poly-A correlates inversely with G+C content, as expected, with the exception of CpG islands, which display an increase in poly-A content corresponding closely to the reduction in observed nucleosome occupancy *in vivo* (**Figure 2A**, middle). This observation is consistent with our previous finding that G+C content and poly-A content are at least partially independent in predicting nucleosome occupancy [22].

## Discussion

The observations presented here indicate that, unlike yeast promoters, which often contain nucleosome-free regions that are hard-coded into the genomic sequence through their intrinsic nucleosome preferences, human promoters and other regulatory sites are, in general, programmed for high nucleosome occupancy. We note that this finding is in contrast to results reported in a recent study [26], which showed that CpG-containing promoter sequence is refractory to nucleosome formation *in vitro*. However, these experiments measured the nucleosome formation potential of only a handful of CpG and non-CpG containing promoters relative to each other: 26 promoter sequences in total, 25 of which have higher than average intrinsic nucleosome occupancy according to our model. As a result, these findings may reflect relative occupancy among CpG promoters, not genome-wide trends. In addition, we and the authors of the aforementioned study note that the positive control used in these experiments, the 601 sequence [27], which forms highly stable nucleosomes *in vitro*, conforms to the standard definition of a CpG island [28]. We note that, on average, poly-A content does increase at exactly the position in CpG promoters at which there is a reduction in *in vivo* nucleosome occupancy, raising the possibility that the depletion may be at least partially caused by intrinsic nucleosome sequence preferences that are not captured by our model, rather than by RNA Pol II [6], [7]. An *in vitro* nucleosome assembly map of the human genome should resolve this issue, and would also allow refinement of our model.

We propose that high intrinsic nucleosome occupancy of regulatory sequences in human serves several purposes. First, given that most human regulatory sites act in a cell-type specific manner, it may be advantageous to keep them masked with nucleosomes unless they are in use, to minimize instances of inappropriate utilization and aberrant transcription from open chromatin. High nucleosome occupancy would also tend to reinforce cooperative interactions between TFs in displacing nucleosomes [29], [30], potentially providing an additional level of specificity in gene regulation.

It may also be important that nucleosomes are incorporated into active, open chromatin. We note that DNaseI-hypersensitive regions have higher than average *in vivo* nucleosome occupancy, even when both are measured in the same cell type (as seen in CD4+ cells, rightmost panel in **Figure 2C**). As noted above, we cannot rule out ascertainment bias due to differences in accessibility, but we reiterate that since CTCF and GABP sites are clearly nucleosome-depleted in these same data, then at the very least the DNaseI-hypersensitive regions are less depleted on the whole than are CTCF and GABP sites, and must therefore contain at least some nucleosomes. There are several additional lines of support that these regions are occupied by nucleosomes even in cell types in which they are active. First, because DNaseI can cleave both linker and nucleosome-associated DNA [31], [32], nucleosomes and DNaseI-hypersensitivity are not mutually exclusive. Second, specific histone marks are enriched at and characteristic of promoters and enhancers [14], indicating that nucleosomes are present at these loci. Third, there are numerous examples in which the activity of regulatory regions is associated not with nucleosome clearance, but rather with rearrangement of nucleosomes, and/or displacement from small regions [33], [34], [35], as appears to be the case for CTCF- and GABP-bound regions (**Figure 2B**). Fourth, there is evidence that nucleosomes can be included in complexes formed by TFs binding to enhancers [36], and it has been proposed that the inclusion of nucleosomes in the architecture of regulatory sites could enable long-range interactions among TF binding sites, because TFs (such as CTCF and GABP) that constrain the positions of adjacent nucleosomes also constrain the relative accessibility of TF binding sites in the same DNA [25]. Fifth, and finally, both chromatin and regulatory complexes at regulatory sites are dynamic on timescales as short as minutes [37], [38], raising the possibility that, within a homogenous culture, at a given time and at a given regulatory locus, different cells may have different profiles of occupancy by transcription factors, nucleosomes, and/or RNA polymerase.

In summary, we propose that high intrinsic nucleosome occupancy of regulatory regions can provide multiple mechanisms for achieving specificity of gene regulation in large genomes, and that it may in fact be a hallmark of genome organization in complex eukaryotes. Moreover, we postulate that the strong influence of G+C content on intrinsic nucleosome occupancy provides at least a partial explanation for the pervasive occurrence of high G+C content regions on diverse scales in a variety of genomes, and its correlation with promoters, genes, and regulatory sites in human and other organisms.

## Methods

We predicted the average intrinsic nucleosome occupancy [1] across each basepair of the human genome (build hg18). We normalized both the nucleosome occupancy predictions and the *in vivo* nucleosome profiles from human CD4+ T-cells [8] at each base pair by taking the log_2_(average basepair score/mean genomic score). We then set the genomic average to zero by subtracting the new mean from each base pair for both intrinsic (i.e. model predictions) predictions and *in vivo* (i.e. CD4+) data. We defined proximal promoters as [−150, 0] from the transcription start site, using the 32,043 promoters in dbTSSv6 [39]. We used 5,015 promoters with well-defined transcription start sites from yeast defined in [20]. CpG island annotations were downloaded from the UCSC genome browser (hg18). We classified proximal promoters as CpG-containing if they overlapped a UCSC CpG island annotation and non-CpG otherwise. For TFBSs and putative regulatory sequences, we restricted the analyses to the ENCODE regions, in order to make direct comparisons among the data sets. We used 778 FAIRE peaks from human fibroblasts [3], 821 DNaseI sites and 118 p300 sites from HeLa cells [14], and 1,213 DNaseI sites from CD4+ T-cells [15] that did not overlap a promoter ([−1,000, 0] from the TSS), all within ENCODE regions. We used 103 GABP, 39 NRSF (monoclonal antibody), 42 NRSF (polyclonal antibody), and 43 SRF ChIP-seq peaks from Jurkat cells [11], 888 STAT1 ChIP-seq peaks from HeLa cells [12], and 206 CTCF ChIP hits from CD4+ T-cells [13], all within ENCODE regions. We used only CTCF sites that contain a CTCF binding sequence [40], to select for those in which the DNA-binding activity of CTCF is utilized.
